# Guidance on use of race, ethnicity, and geographic origin as proxies for genetic ancestry groups in biomedical publications

**DOI:** 10.1016/j.ajhg.2024.03.003

**Published:** 2024-03-12

**Authors:** W. Gregory Feero, Robert D. Steiner, Anne Slavotinek, Tiago Faial, Michael J. Bamshad, Jehannine Austin, Bruce R. Korf, Annette Flanagin, Kirsten Bibbins-Domingo

**Affiliations:** 1 Maine-Dartmouth Family Medicine Residency, Augusta, ME, USA; 2 *JAMA*, Chicago, IL, USA; 3 University of Wisconsin School of Medicine and Public Health, Madison, WI, USA; 4 *Genetics in Medicine*, New York, New York, USA; 5 Department of Pediatrics, Cincinnati Children’s Hospital Medical Center, University of Cincinnati College of Medicine, Cincinnati, OH, USA; 6 *American Journal of Medical Genetics*, Hoboken, NJ, USA; 7 *Nature Genetics*, San Francisco, CA, USA; 8 Departments of Pediatrics and Genome Sciences, University of Washington, Seattle, WA, USA; 9 *Human Genetics and Genomics Advances*, Cambridge, MA, USA; 10 Departments of Psychiatry and Medical Genetics, University of British Columbia, Vancouver, BC, Canada; 11 *Journal of Genetic Counseling*, Vancouver, BC, Canada; 12 Department of Genetics, University of Alabama at Birmingham, Birmingham, AL, USA; 13 *American Journal of Human Genetics*, Cambridge, MA, USA; 14 *JAMA* and the JAMA Network, Chicago, IL, USA

## Main text

In March 2023, the National Academies of Sciences, Engineering, and Medicine (NASEM) released a consensus study report titled *Using Population Descriptors in Genetics and Genomics Research*.^1^ Sponsored by the US National Institutes of Health, the report is more than a discussion of the use of terminology; the authors of the NASEM report suggest a tectonic shift away from current models that use race, ethnicity, and geographic origin as proxies for genetic ancestry groups (i.e., a set of individuals who share more similar genetic ancestries) in genetic and genomic science. The recommendations are rooted in evidence that genetic variation in individuals falls, in general, on a continuum of variation not captured well by existing population descriptors and that the ongoing use of such descriptors as analytical variables jeopardizes the scientific validity of research.^2^ Furthermore, the authors of the NASEM report point out that current scientific practices can sometimes perpetuate harmful typological thinking about individuals, including racism.

Shifting genetic and genomic science away from the pervasive and long-standing use of race, ethnicity, and geographic origins as tools for subdividing people presumed to have greater shared genetic ancestry will not be easy. The proposed changes have implications for genetic and genomic study design, data analysis, and results interpretation and would require sustained support on the part of various stakeholders. The report offers a nuanced strategy to facilitate the shift, outlining a framework for behavior change for the field of human genetics founded on principles of respect, beneficence, equity and justice, validity and reproducibility, and transparency and replicability. These principles underlie the remaining ^3^ domains of the framework that include requisites for sustained change, specific guidance for the selection and use of population descriptors in genetics and genomics research, and strategies for implementation and accountability. A total of 13 recommendations are detailed in the report, each related to one of these domains. The recommendations encompass a wide variety of stakeholders in science from study participants to researchers to funders to biomedical journal editors.

Given the breadth of influence of genetic and genomic science on all areas of biomedicine, the consensus report’s implications extend beyond the genetics and genomics research community to include all researchers who use genetic and genomic data as well as a broader audience. If the recommendations of the report are embraced only by genetics and genomics researchers but not more broadly, breakthrough discoveries may have scientific underpinnings that treat individuals and populations differently from how the remainder of biomedicine treats them. This could have unexpected or negative implications for the translation of genetic and genomic discoveries to the care of individuals and populations. The charge to the consensus study committee specifically excluded “examining the use of race and ethnicity in clinical care” and “examining the use of race and ethnicity in biomedical research generally (non-genetic and genomic research),” thereby focusing the report narrowly on genetic and genomics research up to the point of clinical integration.^3^ The consensus report lacks concrete guidance on how to bridge potential gaps created between genetic and genomic science and the rest of biomedicine should the recommendations gain wide adoption, though further work is underway.^4^ As journal editors, we believe that it is incumbent on us to help bridge any emerging gap, thereby ensuring both the scientific accuracy and interpretability of journal content.

Biomedical journals have a unique role in the translation and dissemination of genetic and genomic science to readers including researchers, clinicians, media, and the general public. The consensus report recognizes research journals as elements of the ecosystem of genomic science with a responsibility to help implement the report’s recommendations.^1^ Specifically, recommendation 9 suggests journals should “offer tools widely to their communities to facilitate the implementation of these recommendations,” and the report includes an appendix with a checklist providing authors and reviewers guidance on the appropriate use of population descriptors in manuscripts. Recommendation 12 suggests that journals “should ensure that policies and procedures are aligned with these recommendations and invest in developing new strategies to support implementation when needed.”

We journal editors concur broadly with the consensus study recommendations that population descriptors such as race, ethnicity, and geographic origin should no longer be used as proxies for genetic ancestry groups in genomic science. We also recognize that this is just one dimension of the use of population descriptors in clinically relevant research, and that drawing a distinction for requirements for genetic and genomic research and the rest of biomedicine could prove challenging. For example, the authors of the NASEM report recognize that racism can be considered a social determinant of health that can have effects on health outcomes far larger than those caused by shared genetic variation.^5^ Continued use of descent-associated population descriptors (e.g., race, ethnicity) to define population groups that have historically experienced health and health care disparities may be necessary for genetics and genomics research exploring, for example, genomic health care access disparities. However, their use in this research may cause confusion outside of the genomics community regarding these descriptors’ lack of utility as proxies for shared genetic ancestry groups. We concur with the authors of the NASEM report that changes in thinking and behaviors regarding population stratification by shared genetic characteristics and the use of population descriptors will be a gradual process.

Biomedical journals have made substantial strides in improving guidance provided to authors and reviewers regarding language for population descriptors. However, more could be done to guide authors and reviewers toward acceptable use of data labeled previously by population descriptors. Drawing on the ethical principles underpinning the consensus study and previous work, we propose the following precepts to authors and reviewers for manuscripts that include use of population descriptors as proxies, at least in part, for genetic ancestry groups^2^^,^^6^^,^^7^:

1. Terminology used for population descriptors should be accurate, respectful, and adhere to current guidance in biomedical science.^6^^,^^7^
2. Self-identified race and ethnicity should not be used as proxies for genetic ancestry groups or to represent the genetic diversity of study participants and are not recommended for use as analytic variables.
3. The rationale for using population descriptors should be provided and justified in the methods section.
4. Description of how participants were classified with population descriptors, including self-identification for racial and ethnic categories, should be included in the methods section.
5. The methods section should clearly explain how the selected descriptors were operationalized in participant accrual, study design, data analysis, and data interpretation.
6. Wherever possible, all genetic ancestry groups should be included in analyses; if any groups are excluded, this should be adequately justified scientifically.
7. The discussion and conclusions sections should explain how the use of populations categorized by population descriptors influenced the interpretation of the study data and conclusions drawn, including if this is unknown.
8. The discussion and conclusions sections should explain how stratified analyses using the selected population descriptors affect the generalizability of the study findings to other populations.
9. When using legacy datasets, it may not be possible to derive and use more accurate measures of genetic ancestry groups. In these instances, authors should explain the limitations of the dataset and the effects these limitations have on data analysis and interpretation.
10. Generally, titles and conclusions sections should avoid inclusion of race and ethnicity as population descriptors for genetic ancestry groups. Such usage may be appropriate for studies in historically isolated populations or those about health care disparities where the social constructs of race and ethnicity are important contributors to outcomes.

Wide adoption of these recommendations would be an important step toward shifting biomedical science away from the use of population descriptors that perpetuate inaccurate assumptions regarding genetic ancestry. Furthermore, adoption of this guidance would not be proscriptive as authors would have an opportunity to defend the validity of their scientific use of population descriptors. Finally, we hope that these practices will be embraced and generalized beyond genetics and genomics, which should help prevent development of a gap between the genetics and genomics research community and the rest of biomedicine. Some researchers, authors, peer reviewers, editors, and journal readers may view these recommendations as burdensome and unnecessary, while others may think them insufficient. It is our intention that these recommendations spark dialogue and actions that improve use and reporting of population descriptors in all of biomedicine.
